# Fenwickism and How to Fight It

**Published:** 1918-12-07

**Authors:** 


### FENWICKISM AND HOW TO FIGHT IT.

The Fenwick brood are out for mischief, and it would be a mistake to assume that their capacity for destruction is negligible. German raiders sent many British merchantmen to the bottom at a time when German first-class battleships lay hiding in the Kiel Canal. The war has taught us that the more desperate the plight of the enemy the more unscrupulous may become his methods of offence. Within the past month the Fenwick offensive has deprived Irish nurses of a thousand pounds. Their objective is not the nurse, but the College of Nursing, and in order, if possible, to wreck the College they do not scruple to involve the rank and file of the profession, if need be, in ruin. The Fenwick press, it may be observed, do not advertise this thousand pounds deprivation. They cannot condescend to explain to the woman in harness why her prospect of support in old age or infirmity should be blighted in order that a petty faction, in which she has no interest, might survive. It is obvious that every dart levelled at the College, whatever damage it may do, must also injure the wreckers, for, sooner or later, the nurse will perceive that she, and she only, is the ultimate sufferer.

### A Pneumatic "Corporation."

In the meantime much harm may be done. These women may be insignificant in number, but, as Sir Arthur Stanley points out, they have assumed high-sounding titles, and they address their attention to an uninformed public. Miss Isabel Macdonald's "urgent request of members of this Corporation, and of organised societies of nurses affiliated to it," might mean a membership of a million. "This Corporation" might, according to the description, correspond with that of the Bank of England, or, *per contra*, with poor old Falstaff's. What is it, in actual fact? We who know realise that it is nothing more than a wind instrument, but the British public are uninformed. It does not occur to them that the Bedford-Fenwick "Corporation" is pneumatic.

But even a drum may do serious damage. It is on historical record that whole armies, not to say whole nations, have been betrayed by a drum, and, therefore, this faction must be fought. The cause of the nurse is too sacred to be left to chance, especially when the traitors are clad in professional uniform.

The question is, How can Fenwickism be fought and conquered? There is one only way, and that is by propaganda. Spread the light. In my opinion, be it sound or otherwise, College propaganda has been weak. The College has behind it unlimited power. But it is latent. It has not been exerted or exploited. The cause of the British nurse has not been demonstrated in anything approaching adequate degree to the British public. "To those who know," says Sir Arthur Stanley, "the protest carries no weight." Just so. But who are those who know? They are a comparative few. The man in the street, the British citizen, is wholly unable to distinguish between the College of Nursing and Miss Isabel Macdonald's pneumatic "Corporation." And yet Miss Isabel Macdonald, standing behind this mystery, is, without any manner of doubt, depriving the women who have helped to win the war of any form of substantial reward.

### A Forward Policy for the College.

The only way for the College of Nursing to fight this brood is by adopting a forward policy. Let the nurse see, and also let the nation and the Empire see, that the College is the working nurse's champion. The Fenwicks are bankrupt of policy. For thirty years they have lived, not on a policy, but on a "stunt." That "stunt" is State Registration. State Registration will probably arrive some day; but when it comes there will be no accompanying endowment. The College stands for Registration, but for vastly more. It stands for democratic reform, for the economic betterment of the rank and file, for shorter hours of labour, better pay, and a provision for old age. It stands for greater efficiency and higher education.

### The British Public Waiting.

But these potential benisons have not been preached from the housetop, and hence the power of the raiders to carry on their piratical warfare. The only way to kill Fenwickism is by a vigorous offensive. There must be no hesitancy, no apparent weakness. The nurse must feel and realise that the College is her out-and-out champion. When she does, there will be a membership not of ten thousand, but of ten times ten thousand. And the British public will respond. The British public is waiting to respond.

On the other hand, if there is a lack of championship, of wholehearted, uncompromising, vigorous propaganda, of determined fighting energy, not all the printers in the world may save the College from being regarded as too dilatory and deliberate in its progressive developments.

IERNE.

